# Neural Network‐Based Study for Rice Leaf Disease Recognition and Classification: A Comparative Analysis Between Feature‐Based Model and Direct Imaging Model

**DOI:** 10.1002/fsn3.71350

**Published:** 2025-12-19

**Authors:** Farida Siddiqi Prity, Mirza Raquib, Saydul Akbar Murad, Md. Jubayar Rafi, Md. Khairul Bhuiyan, Anupam Kumar Bairagi

**Affiliations:** ^1^ Department of Computer Science and Engineering Netrokona University Netrokona Bangladesh; ^2^ Department of Computer Science and Engineering International Islamic University Chittagong Chattogram Bangladesh; ^3^ School of Computing Sciences and Computer Engineering University of Southern Mississippi Hattiesburg Mississippi USA; ^4^ Department of Computer Science and Engineering Daffodil International University Dhaka Bangladesh; ^5^ Department of Electrical & Electronic Engineering BRAC University Dhaka Bangladesh; ^6^ Computer Science and Engineering Discipline Khulna University Khulna Bangladesh

**Keywords:** Artificial Neural Network, disease, Extreme Learning Machine, Feature Extraction Algorithm, rice

## Abstract

Rice leaf diseases significantly reduce productivity and cause economic losses, highlighting the need for early detection to enable effective management and improve yields. This study proposes Artificial Neural Network (ANN)‐based image‐processing techniques for timely classification and recognition of rice diseases. Despite the prevailing approach of directly inputting images of rice leaves into ANNs, there is a noticeable absence of thorough comparative analysis between the Feature Analysis Detection Model (FADM) and the Direct Image‐Centric Detection Model (DICDM), specifically when it comes to evaluating the effectiveness of Feature Extraction Algorithms (FEAs). Hence, this research presents initial experiments on the Feature Analysis Detection Model, utilizing various image Feature Extraction Algorithms, Dimensionality Reduction Algorithms (DRAs), Feature Selection Algorithms (FSAs), and Extreme Learning Machine (ELM). The experiments are carried out on datasets encompassing 3829 original rice leaf images across six classes (bacterial leaf blight, brown spot, leaf blast, leaf scald, sheath blight rot, and healthy leaf). A Direct Image‐Centric Detection Model is established without the utilization of any FEA, and the evaluation of classification performance relies on different metrics. Ultimately, an exhaustive contrast is performed between the achievements of the Feature Analysis Detection Model and the Direct Image‐Centric Detection Model in classifying rice leaf diseases. The results reveal that the highest performance is attained using the Feature Analysis Detection Model. We have also applied Gradient‐weighted Class Activation Mapping (Grad‐CAM) for visual interpretability of the model's predictions. The adoption of the proposed Feature Analysis Detection Model for detecting rice leaf diseases holds excellent potential for improving crop health, minimizing yield losses, and enhancing the overall productivity and sustainability of rice farming.

## Graphical abstract

This study proposes a NN‐based framework for automated recognition and classification of rice diseases using leaf imagery. Feature extraction techniques such as texture analysis, GLCM, GLDM, FFT, and DWT extract critical image characteristics, while dimensionality reduction (PCA, KPCA, Sparse AE, Stacked AE) and feature selection (ANOVA F‐measure, Chi‐square test, RF) optimize classification. The framework uses ELM to categorize bacterial leaf blight, brown spot, leaf blast, leaf scald, sheath blight rot, and healthy leaves, achieving early and precise disease identification.

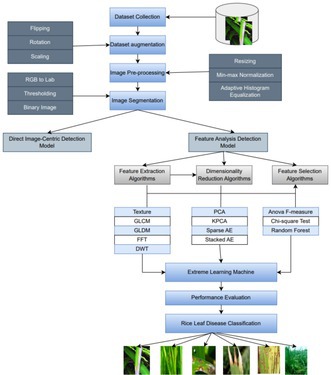

## 1 Introduction

Rice is one of the most vital staple crops worldwide, providing sustenance for over 3.5 billion people, particularly in Asia, Africa, and Latin America (Bari et al. 2021). In Asia alone, where rice forms the backbone of diets, it accounts for over 90% of global production and consumption (Christou and Twyman 2004). This crop contributes to the primary caloric intake for 60% of the Asian population, underscoring its irreplaceable role in regional food security (Dordas 2008). Globally, rice provides approximately 20% of human dietary energy and is an essential source of livelihood for millions of smallholder farmers (Daniya and Vigneshwari 2022a).

Despite its critical importance, rice production faces significant threats from various leaf diseases, which collectively result in severe crop losses annually (Sharma and Singh 2022; Liu et al. 2023). These diseases, including bacterial blight, rice blast, brown spot, and tungro, can cause staggering yield losses (Liang et al. 2019). For example, rice blast alone is known to destroy between 10% and 30% of the global rice crop each year, contributing to annual economic losses of approximately $5 billion (Jiang et al. 2023). Overall, rice leaf diseases contribute to an estimated 15%–30% reduction in global rice yields, equating to millions of tons of rice lost and billions in economic damage each year (Feng et al. 2020). Such losses have dire implications for food security, affordability, and accessibility in rice‐dependent regions, particularly in Asia, where rice diseases threaten the stability of food systems and the livelihoods of vulnerable farming communities (Ganesan and Chinnappan 2022; Verma and Dubey 2021).

The importance of accurately detecting rice leaf diseases cannot be overstated. Early identification enables timely intervention, reducing the need for extensive pesticide applications and limiting the spread of infections to healthy plants (Quach et al. 2023; Al‐Gaashani et al. 2023; Goluguri et al. 2021). In regions highly dependent on rice, such as Asia and Africa, where small‐scale farmers rely on high yields to sustain their livelihoods, rapid and precise disease detection is critical (Kukana 2020; Gianessi 2014). Timely detection and intervention safeguard yield volumes and enhance the overall quality of rice, reducing reliance on costly agrochemicals that can disrupt ecosystems (Singh et al. 2019). Furthermore, early disease management helps conserve resources, supports sustainable farming practices, and ultimately strengthens food security at both national and global levels.

Traditional approaches to rice disease detection, such as manual inspection by experts, are time‐consuming, labor‐intensive, and prone to human error (Liu et al. 2020; Zarbafi and Ham 2019; Han et al. 2014; Guo et al. 2010; Feng et al. 2020). To overcome these challenges, recent advances in digital imaging and artificial intelligence (AI) have inspired the development of automated models for disease recognition (Prity et al. 2023, 2024). In particular, machine learning and deep learning frameworks such as Convolutional Neural Networks (CNNs) and transfer learning have been applied to rice leaf disease classification (Wu and Feng 2018; Kujawa and Niedbała 2021; Escamilla‐García et al. 2020). While these methods have achieved encouraging results, several critical limitations persist. Many studies rely on small or imbalanced datasets (Samborska et al. 2014; Zorzetto et al. 2000; Smrekar et al. 2009; Verma and Dubey 2017; Azim et al. 2021; Yao et al. 2009; Islam and Mazumder 2019; Ghyar and Birajdar 2017; Saputra et al. 2020; Matin et al. 2020; Lu et al. 2017; Rahman et al. 2020; Latif et al. 2022; Simhadri and Kondaveeti 2023), which reduce model generalization. Some works applied only a limited set of feature extraction techniques (Verma and Dubey 2017; Azim et al. 2021; Yao et al. 2009; Islam and Mazumder 2019; Ghyar and Birajdar 2017; Saputra et al. 2020) or neglected important preprocessing steps (Ghyar and Birajdar 2017; Rahman et al. 2020), thereby weakening robustness. Others (Verma and Dubey 2017; Azim et al. 2021; Yao et al. 2009; Islam and Mazumder 2019; Ghyar and Birajdar 2017; Saputra et al. 2020; Matin et al. 2020; Lu et al. 2017; Rahman et al. 2020; Latif et al. 2022; Simhadri and Kondaveeti 2023; Rice Leaf Diseases Dataset 2023; Narin et al. 2021; Chen et al. 2020; Bijoy et al. 2024; Pizer et al. 1987) employed CNN‐based models but without any Overfitting Reducing Methods (ORM) such as cross‐validation or early stopping, leading to overfitting and reduced adaptability in real‐world scenarios. Collectively, these issues highlight a key research gap: existing studies either focus narrowly on handcrafted feature extraction or depend solely on direct image‐centric approaches, without systematically comparing their strengths and weaknesses. Furthermore, the absence of advanced dimensionality reduction and feature selection methods has led to redundant features, computational inefficiency, and limited interpretability. Therefore, there is a pressing need for a unified and comprehensive framework that addresses these shortcomings by rigorously evaluating both feature‐based and direct image‐centric models using balanced datasets, multiple algorithms, and robust validation strategies.

Our study proposes a comprehensive and refined approach to address these limitations. The main contributions of this study are:

- Employing diverse Feature Extraction Algorithms (Texture analysis, Gray Level Co‐occurrence Matrix (GLCM), Gray Level Difference Matrix (GLDM), Fast Fourier Transform (FFT), and Discrete Wavelet Transform (DWT)) to capture complementary disease‐related features.
- Applying Dimensionality Reduction Algorithms (Principal Component Analysis (PCA), Kernel Principal Component Analysis (KPCA), Sparse Autoencoder (Sparse AE), and Stacked Autoencoder (Stacked AE)) to refine the feature space and improve classification performance.
- Utilizing Feature Selection Algorithms (Anova F‐measure, Chi‐square Test, and Random Tree (RF)) to optimize efficiency and eliminate redundancy.
- Developing a comparative framework between Feature Analysis Detection Model and Direct Image‐Centric Detection Model, systematically assessing the role of feature engineering in rice leaf disease detection.
- Incorporating early stopping and 10‐fold cross‐validation to mitigate overfitting and ensure robust performance evaluation.
- Applying Grad‐CAM for visual interpretability, confirming that the model attends to disease‐affected regions.

The structure of this paper is as follows: Section 2 presents a literature review, summarizing prior research on rice leaf disease detection methods. Section 3 outlines the methodology, covering data collection, preprocessing, and model training techniques. In Section 4, we discuss our findings, with an emphasis on model performance metrics. Finally, Section 5 concludes the paper with a summary of results and directions for future research.

## 2 Previous Works

Recently, many authors have incorporated AI techniques to classify rice disease. Rice disease detection using AI is mainly based on two strategies proposed till now: Feature Analysis Detection Model and Direct Image‐Centric Detection Model. The FADM in rice disease detection involves extracting relevant features from the leaf image to represent its content, such as specific patterns, textures, shapes, or other visual attributes essential for identifying and distinguishing the diseases. In contrast, the DICDM bypasses the feature extraction step and directly uses the raw pixel data for analysis and interpretation. While the Feature Analysis Detection Model focuses on capturing specific patterns and attributes, the Direct Image‐Centric Detection Model relies on the entire image for classification and identification purposes.

### 2.1 Overview of Previous Feature Analysis Detection Model of Rice Disease Recognition

Verma et al. presented a Feature Analysis Detection Model for rice disease detection, where the hybrid features are extracted using Discrete Cosine Transform (DCT) (Verma and Dubey 2017). The extracted features are then classified using inverse multi‐quadrics Radial Basis Function (RBF) and Decision Tree, significantly improving the recognition efficiency from 16.67% to 83.34%. Azim et al. developed a model using GLCM and Local Binary Pattern (LBP) as textural feature descriptors to detect diseases in rice (Azim et al. 2021). XG Boost and Support Vector Machine (SVM) achieved an accuracy of 86.58% for disease classification using this approach. Yao et al. utilized GLCM and SVM to detect and classify rice diseases (Yao et al. 2009). Islam et al. employed DWT for multi‐resolution analysis of rice disease images, followed by classification using an ensemble of linear classifiers with the Random Subspace Method (RSM) (Islam and Mazumder 2019). Ghyar et al. utilized GLCM to classify rice diseases (Ghyar and Birajdar 2017). The classification was performed using SVM and Artificial Neural Networks. Saputra et al. (Saputra et al. 2020) proposed using GLCM as a feature extraction method for text analysis in classifying rice leaf disease images. The classification was done using the K‐Nearest Neighbor (KNN) algorithm, which achieved an accuracy of 65.83%. Table 1 represents the summarized findings of various related works focused on rice disease detection, utilizing Feature Analysis Detection Models to highlight key methods, results, and limitations.

**TABLE 1:** Summary of related works for rice disease detection using feature analysis detection model.

Paper	Dataset	Description of related works	Results	Limitation
Verma and Dubey (2017)	Six diseases: 180 images	DCT RBF Decision Tree	Accuracy 83.34%	Dataset is tiny Use only one FEA No DRA No FSA No ORM
Azim et al. (2021)	Three diseases: 120 images	GLCM LBP XG Boost SVM	Accuracy 86.58%	Dataset is tiny Use only one FEA Focus on only four diseases No DRA No FSA No ORM
Yao et al. (2009)	Three diseases: 216 images	GLCM SVM	Accuracy 97.2%	Dataset is tiny Use only one FEA Focus on only three diseases No DRA No FSA No ORM
Islam and Mazumder (2019)	Five diseases: 135 images	DWT RSM	Accuracy 95%	Dataset is tiny Use only one FEA Focus on only three diseases No DRA No FSA No ORM
Ghyar and Birajdar (2017)	Two diseases: 80 images	GLCM Genetic Algorithm SVM ANN	Accuracy 92.50%	Dataset is tiny Use only one FEA No image contrast method No DRA No ORM
Saputra et al. (2020)	Three diseases: 120 images	GLCM KNN	Accuracy 65.83%	Dataset is tiny Use only one FEA Focus on only three diseases No DRA No ORM

### 2.2 Overview of Previous Direct Image‐Centric Detection Model of Rice Disease Recognition

Martin et al. applied the AlexNet technique to identify three prevalent rice diseases, achieving an impressive accuracy of 99% (Matin et al. 2020). Lu et al. introduced a novel method for rice disease identification based on deep Convolutional Neural Networks (CNNs) (Lu et al. 2017). Rahman et al. developed a CNN model for the classification of eight categories of rice leaf diseases (Rahman et al. 2020). Latif et al. introduced a Deep Convolutional Neural Network (DCNN) transfer learning‐based approach to accurately detect and classify rice leaf disease (Latif et al. 2022). Simhadri et al. employed a transfer learning approach utilizing 15 pre‐trained CNN models to identify rice leaf diseases automatically (Simhadri and Kondaveeti 2023). Table 2 represents the summarized findings of various related works focused on rice disease detection, utilizing Direct Image‐Centric Detection Models to outline key methods, results, and limitations.

**TABLE 2:** Summary of related works for rice disease detection using direct image‐centric detection model.

Paper	Dataset	Description of related works	Results	Limitation
Matin et al. (2020)	Three diseases: 120 images	AlexNet	Accuracy 99%	Dataset is tiny Focus on only three classes No DRA No FSA
Lu et al. (2017)	Two types of images: 500 images	CNN	Accuracy 95.48%	Dataset is tiny Focus on only two classes No DRA No FSA No ORM
Rahman et al. (2020)	Nine diseases: 1426 images	CNN	Accuracy 93.3%	Dataset is not balanced No image contrast method No DRA No FSA
Latif et al. (2022)	Six diseases: 2167 images	CNN	Accuracy 96.08% Precision 0.9620 Recall 0.9617 Specificity 0.9921 F1‐score 0.9616	Dataset is not balanced No DRA No FSA
Simhadri and Kondaveeti (2023)	Nine diseases: 9074 images	ResNet50 ResNet101 GoogleNet Shufflenet MobileNetV2 Efficientnetb0 DenseNet201 AlexNet Squeeznet Darknet53 InceptionV3 InceptionResnetV2 Xception	Accuracy 99.64% Precision 98.23% Recall 98.21% F1‐Score 98.20% Specificity 99.80%	Dataset is not balanced No DRA No FSA

### 2.3 Critical Gap Analysis and Motivation

While both FADM‐based and DICDM‐based approaches have shown promise, several limitations persist in existing works:

- Small and imbalanced datasets: Many studies, such as Verma and Dubey (2017), Azim et al. (2021), and Yao et al. (2009), relied on very small datasets (80–216 images), which reduce the generalization of trained models. Even larger studies (e.g., Rahman et al. (2020), Latif et al. (2022), Simhadri and Kondaveeti (2023)) often used imbalanced data, which biased classification.
- Limited feature extraction: FADM‐based works often employed only one feature extraction method (e.g., GLCM or DWT), which restricted the richness of feature representation (Verma and Dubey 2017; Azim et al. 2021; Yao et al. 2009; Islam and Mazumder 2019; Ghyar and Birajdar 2017; Saputra et al. 2020).
- Lack of dimensionality reduction and feature selection: Most prior studies did not incorporate PCA, KPCA, or autoencoders to reduce redundancy, nor did they use feature selection algorithms to optimize the feature space. This limited efficiency and risked overfitting.
- Overfitting and generalization issues: Several deep learning‐based DICDM approaches achieved high accuracy on training sets but lacked proper overfitting control (e.g., cross‐validation, early stopping), making them less robust in real‐world scenarios (Rahman et al. 2020; Latif et al. 2022; Simhadri and Kondaveeti 2023).
- Absence of comparative analysis: To date, there has been no systematic comparison between FADM and DICDM within a unified experimental setting using a balanced rice disease dataset.

To address these gaps, the present study integrates multiple Feature Extraction Algorithms, dimensionality reduction techniques, and feature selection methods within the FADM, and contrasts it with a baseline DICDM. By using a balanced dataset of six rice disease classes, applying data augmentation, employing 10‐fold cross‐validation, and incorporating Grad‐CAM for visual interpretability, this research provides a rigorous comparative evaluation. In doing so, it establishes clearer insights into when feature engineering provides significant advantages over direct image‐centric models for rice disease detection.

## 3 Methodology

**FIGURE 1:**
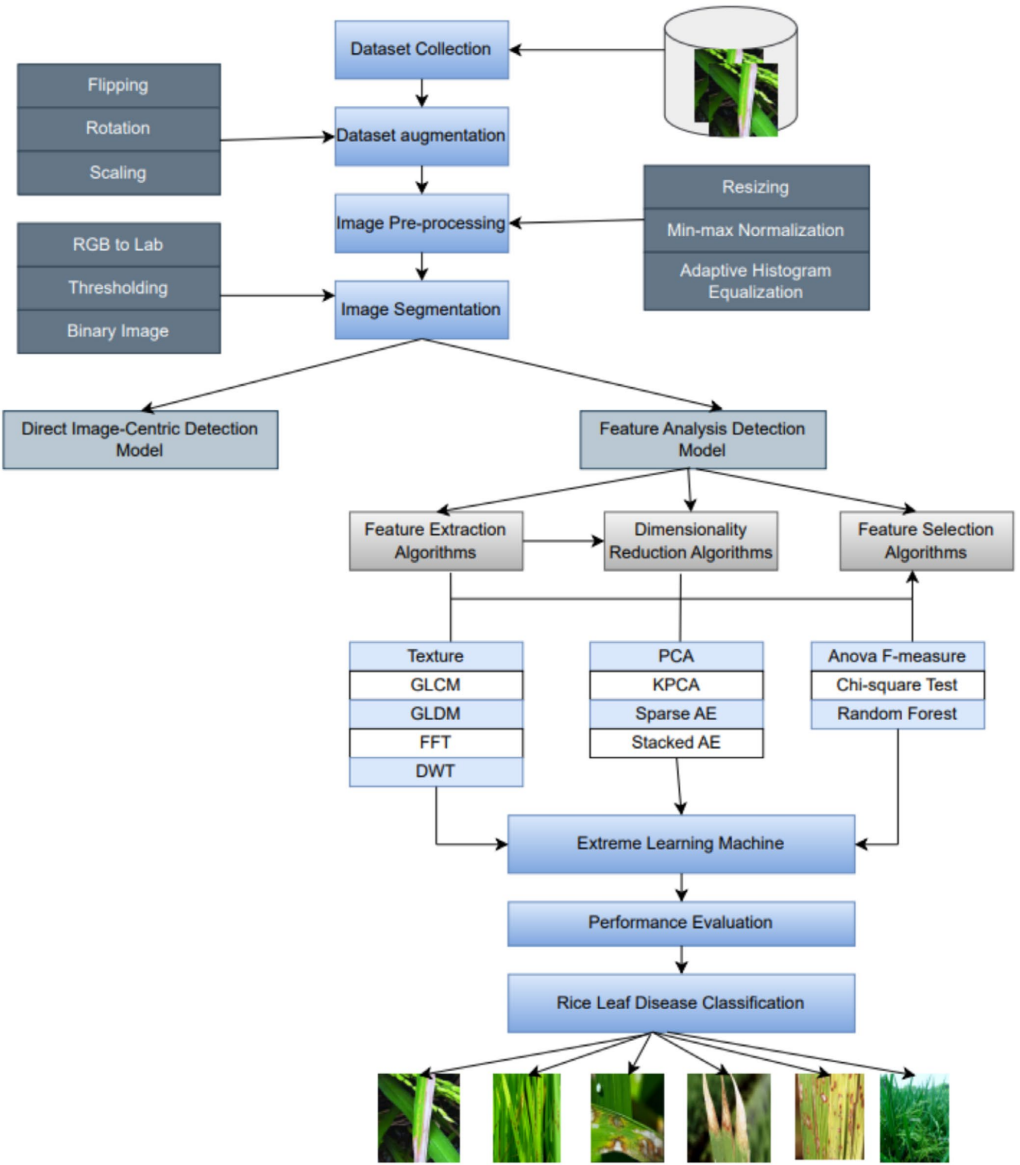
Fundamental process of the proposed study.

This study aims to develop an ANN model for predicting rice diseases. The proposed system comprised different key stages, as illustrated in Figure 1: dataset collection, data augmentation, image pre‐processing, image segmentation, feature extraction, dimension reduction, feature selection, and classification using ANNs. This section labels the paces and strategies employed for segmenting, extracting features, reducing dimensionality, selecting significant features, and recognizing and classifying the diseases of rice plants used in the proposed system.

### 3.1 Dataset Collection

**FIGURE 2:**
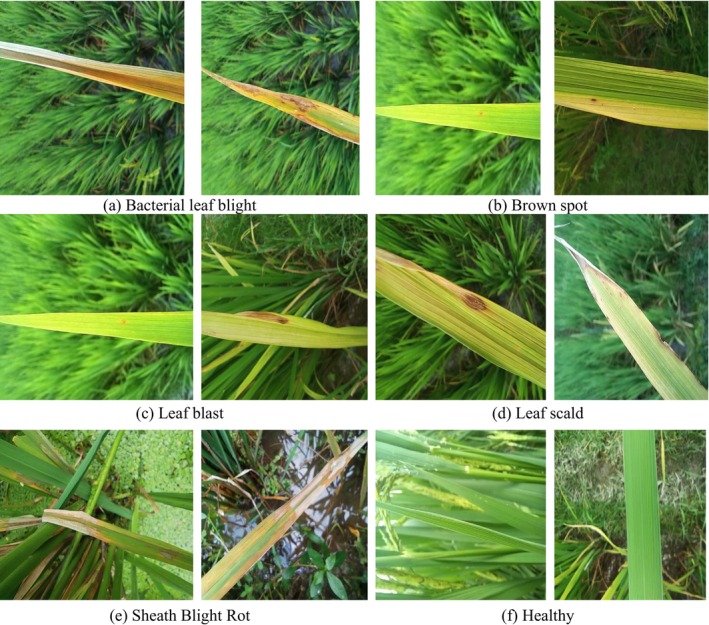
Dataset samples.

In this research, we exclusively utilized rice leaf disease images sourced from the Kaggle dataset (Rice Leaf Diseases Dataset 2023). The dataset contains images classified into six categories: five representing distinct diseases—bacterial leaf blight (636 images), brown spot (646 images), leaf blast (634 images), leaf scald (628 images), and sheath blight rot (632 images)—and one representing healthy leaves (653 images). Figure 2 illustrates representative images from each category, highlighting the characteristic symptoms of the diseases and the normal features of healthy leaves.

### 3.2 Dataset Augmentation

To enhance the dataset's diversity and strengthen model robustness, we applied data augmentation techniques, including flipping, rotation, and scaling, to introduce variations in orientation and size. Horizontal flipping was implemented by using:

(1) $$f\left(x,y\right)=f\left(W-x,y\right)$$

where 𝑊 represents the image width, and each pixel at position $\left(x,y\right)$ is mirrored along the horizontal axis. Rotation was performed by an angle 𝜃, represented by the transformation in:

(2) x′y′=cosθsinθ−sinθcosθxy

where $\left(x,y\right)$ are the original coordinates and $\left(x^{\prime },y^{\prime }\right)$ are the rotated coordinates. Scaling was applied using the transformation shown as follows:

(3) x′y′=sxy

where 𝑠 denotes the scaling factor applied to each coordinate. Through these augmentation techniques, we increased the dataset to 1000 images per class, yielding a more comprehensive range of samples and enhancing the model's capacity to generalize effectively across various conditions.

### 3.3 Image Pre‐Processing

The images were transformed to a standardized dimension of 256 × 256 pixels to ensure consistency across the dataset. Min‐max normalization was applied to scale pixel intensity values, enhancing uniformity and coherence (Narin et al. 2021). This normalization process is defined by:

(4) $$I_{\mathrm{norm}}=\frac{I-I_{\min}}{I_{\max}-I_{\min}}$$

where 𝐼 represents the original pixel intensity, $I_{\min}$ and $I_{\max}$ are the minimum and maximum pixel values, respectively, and $I_{\mathrm{norm}}$ denotes the normalized pixel intensity. Additionally, Adaptive Histogram Equalization (Chen et al. 2020) was implemented to improve local contrast by adjusting the brightness in each image. AHE enhances the visibility of finer details by locally modifying the histogram for sub‐regions within the image, effectively amplifying brightness and improving feature clarity.

### 3.4 Image Segmentation

Image segmentation is crucial in accurately identifying a leaf image's diseased portion. The Red Green Blue (RGB) image is initially transformed into the Lab color space, representing color by three distinct values: L*, a*, and b*. This transformation can be mathematically represented using:

(5) $$\left\{\begin{array}{c} L^{*}\\ a^{*}\\ b^{*} \end{array}\right.=f\left\{\begin{array}{c} R\\ G\\ B \end{array}\right.$$

where 𝑓denotes the conversion function that translates the RGB color values to the Lab color space. Among these, the a* component captures the range from green to red, making it essential for segmenting the affected area. By isolating a* component, the image undergoes a global thresholding process to convert it into a binary image (Bijoy et al. 2024; Pizer et al. 1987). This thresholding can be defined as follows:

(6) $$I_{\text{binary}}\left(x,y\right)=\left\{\begin{array}{c} 1\;\text{if}\;a^{*}\left(x,y\right)>T\\ 0\;\text{if}\;a^{*}\left(x,y\right)\leq T \end{array}\right.$$

where 𝑇 is the threshold value, and $I_{\text{binary}}\left(x,y\right)$ represents the binary output for each pixel $\left(x,y\right)$. The resulting binary image is then overlaid onto the original RGB image, effectively delineating the diseased regions with enhanced clarity.

### 3.5 Direct Image‐Centric Detection Model

In the Direct Image‐Centric Detection Model, segmented images are fed directly into the Artificial Neural Network for classification. Each image, resized to 256 × 256 pixels, serves as the input to the ANN, resulting in 65,536 input nodes per training instance. This direct input approach leverages the pixel intensity values from each segmented image, enabling the ANN to analyze and classify disease patterns directly, bypassing the need for prior feature extraction.

### 3.6 Feature Analysis Detection Model

The Feature Analysis Detection Model utilizes feature extraction to identify and classify disease patterns in images through a structured, multi‐stage process. This model is organized into three key stages:

- Feature Extraction Algorithms stage
- Dimensionality Reduction Algorithms stage
- Feature Selection Algorithms stage

#### 3.6.1 Feature Extraction Algorithms Stage

FEAs are computational techniques in machine learning that convert raw data into meaningful features. These features capture essential data aspects and serve as inputs for models, enhancing pattern learning, prediction, and task performance. The proposed study calculates spatial and frequency domain features for rice disease recognition. This study employs five FEAs to accomplish this task: Texture (He et al. 1987), GLCM (De Siqueira et al. 2013), GLDM (Sen et al. 2009), FFT (Duhamel and Vetterli 1990), and DWT (Nason and Silverman 1994).

The Feature Extraction Algorithms utilized in this study included the calculation of 14 statistical features for each image, capturing essential characteristics. These features include: area, mean, standard deviation, energy, median, skewness, entropy, maximum value, minimum value, mean absolute deviation, kurtosis, range, root mean square, and uniformity. These metrics were computed directly from the segmented 256 × 256 images, providing a foundational dataset to describe variations within the images.

Gray Level Co‐occurrence Matrix and Gray Level Difference Matrix features were computed across four orientations: 0°, 45°, 90°, and 135°. For each orientation, 14 features were calculated, yielding a total of 56 features per method (4 orientations ×14 features). These features capture textural properties, contributing to the model's ability to distinguish diseased regions based on directional texture patterns. For instance, the GLCM for an image $f\left(x,y\right)$ is computed as follows:

(7) $$p\left(i,j\right)=\frac{1}{N}\sum_{x=1}^{M}\sum_{y=1}^{M}\delta \left(f\left(x,y\right)=i,f\left(x+\mathrm{dx},y+\mathrm{dy}\right)=j\right)$$

where $p\left(i,j\right)$ represents the probability of pixel intensity 𝑖 co‐occurring with intensity 𝑗 at a specific displacement vector (𝑑𝑥, 𝑑𝑦), and 𝑀 is the total number of gray levels in the image. The Discrete Wavelet Transform decomposes each image into eight distinct sub‐bands, representing various frequency components within the image. For each sub‐band, 14 features were calculated, resulting in a total of 112 features from DWT (8 bands ×14 features). DWT is expressed mathematically as follows:

(8) $$W_{\varphi }\left(a,b\right)=\frac{1}{\sqrt{a}}\int_{-\infty }^{\infty }f\left(t\right)\varphi \left(\frac{t-b}{a}\right)\mathrm{dt}$$

where $W_{\varphi }\left(a,b\right)$ represents the wavelet coefficient, 𝑎 is the scaling factor, 𝑏 is the translation factor, and φ is the wavelet function. Fast Fourier Transform was applied to analyze the frequency domain by converting spatial data into frequency components. The FFT transformation is represented as:

(9) $$F\left(u,v\right)=\sum_{x=0}^{M-1}\sum_{y=0}^{N-1}f\left(x,y\right)\;e^{-2\mathrm{\pi i}\left(\frac{\mathrm{ux}}{M}+\frac{\mathrm{vy}}{N}\right)}$$

where (𝑥, 𝑦) is the pixel intensity at spatial coordinates, and (𝑢) is the frequency component at frequency coordinates (𝑢). Texture Features were also directly calculated from the segmented images. In total, each image in the dataset contributed a comprehensive set of 252 features: 14 features, 56 GLCM features, 56 GLDM features, and 112 DWT features. These features collectively form a robust dataset, encapsulating both spatial and frequency domain information necessary for effective disease recognition and classification.

In the Feature Extraction Algorithms stage, eight distinct phases assess the classification performance of various feature sets, each using an Artificial Neural Network for classification. First, 14 texture features are extracted and classified by the ANN, followed by 56 Gray Level Co‐occurrence Matrix features and 56 Gray Level Difference Matrix features, each classified using the ANN. Additionally, 14 Fast Fourier Transform features and 14 Discrete Wavelet Transform features are separately classified. After the independent extraction of features using Texture, GLCM, GLDM, FFT, and DWT, the resulting vectors were concatenated into a single composite feature vector referred to as “All.” This produced a unified feature representation of 252 dimensions (14 Texture + 56 GLCM + 56 GLDM + 14 FFT + 112 DWT). Moreover, a subset of 126 frequency domain features (comprising FFT and DWT features) and another subset of 126 spatial domain features (combining Texture, GLCM, and GLDM features) are classified individually using the ANN. This approach allows the model to evaluate both isolated and combined feature sets, facilitating a robust assessment of each feature's effectiveness in accurate disease classification.

#### 3.6.2 Dimensionality Reduction Algorithms Stage

Dimensionality Reduction Algorithms simplify datasets by reducing the number of features while retaining relevant information. These algorithms transform data into a lower‐dimensional representation, enhancing efficiency and minimizing overfitting risks. This study employs four Dimensionality Reduction Algorithms: PCA (Abdi and Williams 2010), KPCA (Schölkopf et al. 1997), Sparse AE (Makhzani and Frey 2013), and Stacked AE (Zabalza et al. 2016).

PCA reduces the dimensionality of the feature vector by identifying directions (principal components) that maximize variance in the data. Given a feature vector = [$x_{1},x_{2},x_{3},\ldots .\ldots ,x_{n}$]. PCA transformation is represented by:

(10) $$X^{\prime }=\mathrm{XW}$$

where 𝑊 is the matrix of eigenvectors of the covariance matrix of 𝑋. This study applies PCA to reduce the feature vector from 252 to 70 features.

KPCA extends PCA by applying a non‐linear kernel function to map data into a higher‐dimensional space, then performing linear PCA in that space. The kernel transformation is represented as follows:

(11) $$K_{\mathrm{ij}}=\varphi \left(x_{i}\right).\varphi \left(x_{j}\right)$$

where 𝐾 is the kernel matrix, capturing inner products in the feature space using a non‐linear mapping φ. KPCA reduces the feature vector from 252 to 65 features.

**FIGURE 3:**
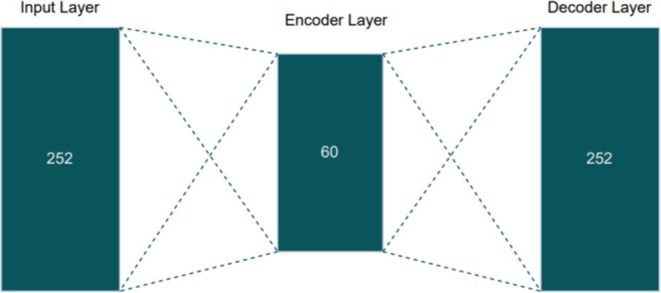
Design of proposed Sparse AE.

The Sparse Autoencoder algorithm compresses high‐dimensional feature vectors into a lower‐dimensional space of 60 features. This architecture includes an input layer for the 252 features, an encoder layer that reduces dimensionality to 60 features, and subsequent decoder layers that reconstruct the input from the encoded representation. The loss function for Sparse AE is given by:

(12) $$L={\left\|X-X^{\prime }\right\|}^{2}+\gamma \sum_{j=1}^{m}\mathrm{KL}\left(p\|p_{j}\right)$$

where ${\left\|X-X^{\prime }\right\|}^{2}$ is the reconstruction error, 𝜆 is the sparsity penalty, 𝐾𝐿 denotes the Kullback–Leibler divergence, 𝜌 is the desired sparsity, and $p_{j}$ is the average activation of hidden unit 𝑗. The specific design of the Sparse AE employed is detailed in Figure 3.

**FIGURE 4:**
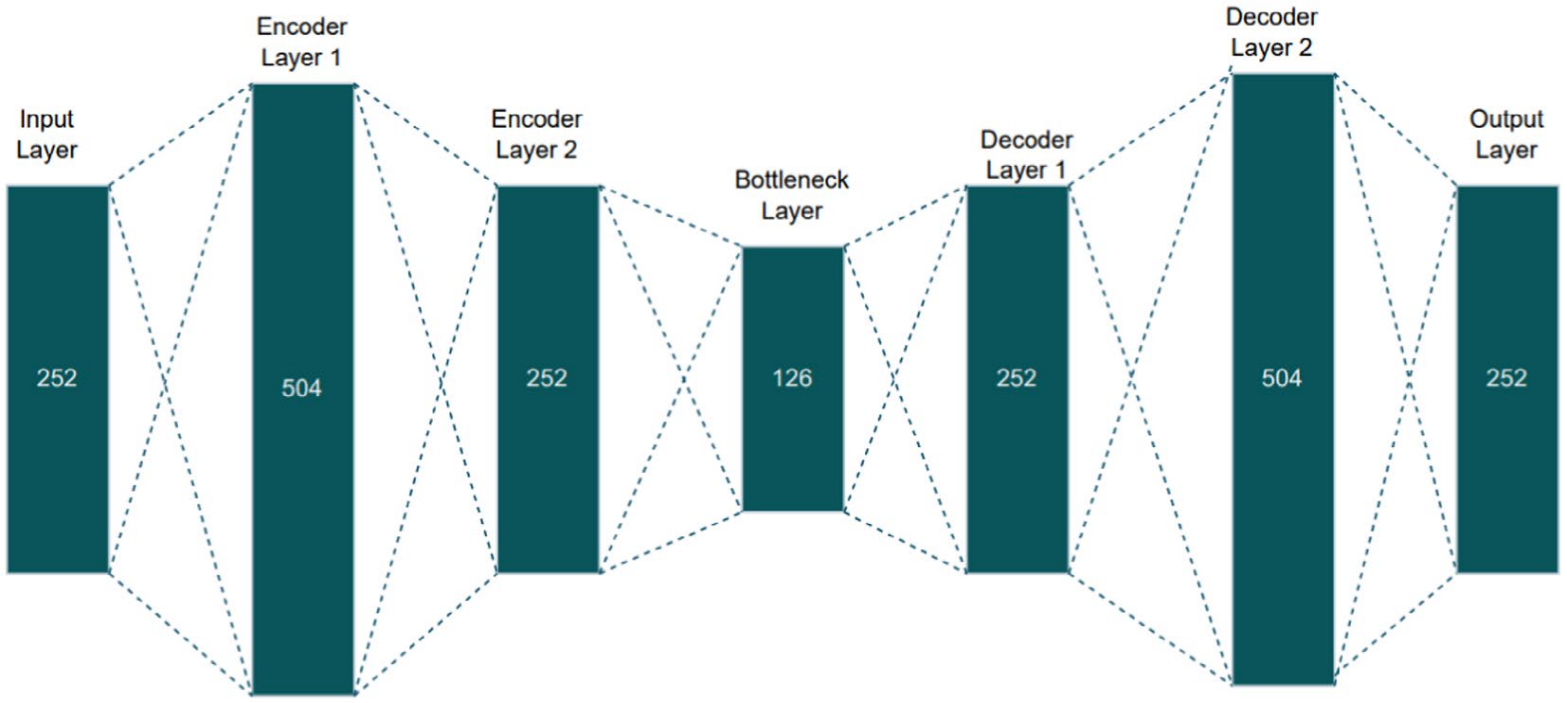
Design of proposed Stacked AE.

The Stacked Autoencoder structure used in this study reduces the dimensionality of the vector from 252 features to 126 features. This architecture includes an input layer with 252 features, followed by two encoder layers that successively compress the data. The output of the second encoder layer serves as the bottleneck, yielding the final feature vector of 126 features. Decoder layers then reconstruct the input data from this bottleneck representation. A detailed depiction of the Stacked Autoencoder's architecture, illustrating the progression of information across layers, is presented in Figure 4. Each encoder layer performs the transformation as follows:

(13) $$H=f\left(\mathrm{WX}+b\right)$$

where 𝑊 is the weight matrix, 𝑏 is the bias, and 𝑓 is the activation function. These reduced feature sets from each DRA are subsequently fed into the Artificial Neural Network for classification.

#### 3.6.3 Feature Selection Algorithms Stage

FSAs are computational techniques used in AI and data analysis to identify and select an array of the most relevant features from more extensive inputs. Feature selection aims to improve model performance by reducing noise, overfitting, and computational complexity. This study employs three Feature Selection Algorithms: Anova F‐measure (Archer et al. 1997), Chi‐square Test (Tallarida et al. 1987), and RF (Belgiu and Drăguţ 2016).

The Anova F‐measure assesses the variance between groups to identify features with strong discriminative power. The F‐score for each feature is calculated as follows:

(14) $$F=\frac{\text{Between}-\text{group variance}}{\text{Within}-\text{group variance}}$$

In this phase, the ANOVA F‐measure identifies 50 out of the original 252 features as significantly discriminative. The chi‐square test evaluates the independence between categorical variables and each feature, calculating how expected frequencies deviate from observed values. The chi‐square score for each feature is computed as follows:

(15) $$X^{2}=\sum_{i=1}^{k}\frac{{\left(O_{i}-E_{i}\right)}^{2}}{E_{i}}$$

where $O_{i}$ and $E_{i}$ are the observed and expected frequencies, respectively, for each category 𝑖. This test identifies 40 significant features from the initial set of 252.

Random Forest selects features based on their importance in the context of tree‐based modeling. The importance score of a feature $x_{i}$ is calculated by observing the mean decrease in impurity (MDI) across all trees in the forest:

(16) $$\text{Importance}\;\left(x_{i}\right)=\sum_{t=1}^{T}\frac{∆I_{T}\left(x_{i}\right)}{T}$$

where 𝑇 is the total number of trees, and $∆I_{T}\left(x_{i}\right)$ is the decrease in impurity in tree 𝑡 due to feature $x_{i}$. Using RF, 35 essential features are selected from the original 252. These selected features from each FSA are subsequently fed into the Artificial Neural Network for classification.

### 3.7 Artificial Neural Network

**FIGURE 5:**
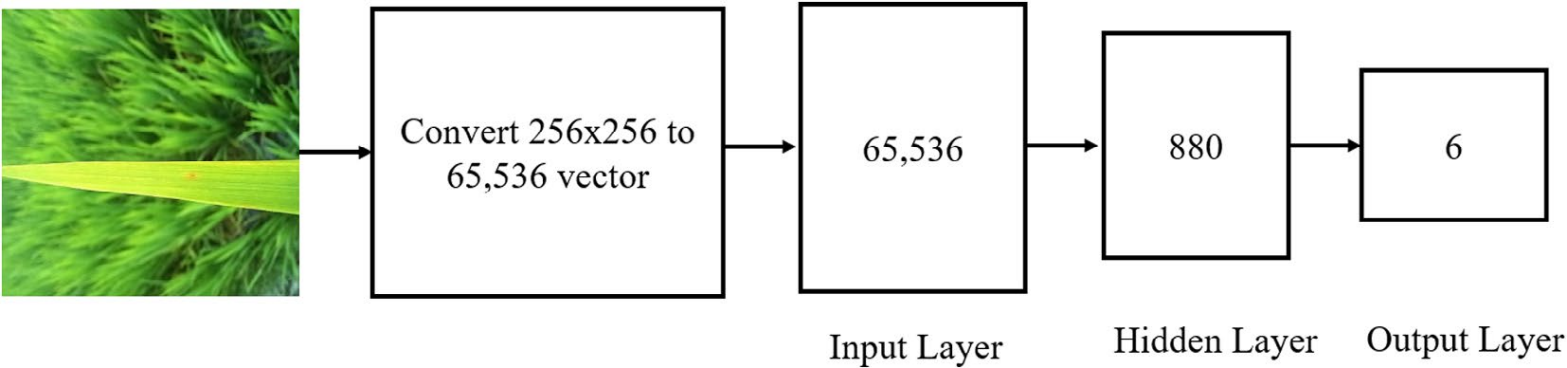
Architecture of ELM for Direct Image‐Centric Detection Model.

Artificial Neural Networks are a powerful tool in modern artificial intelligence, enabling computers to learn and make predictions based on data by emulating, to a certain extent, the information processing mechanisms of the human brain. Their capacity to identify complex patterns and relationships has contributed significantly to advancements across numerous applications, establishing ANNs as a foundational technology in today's technological landscape (Gupta 2013). This research specifically employs the Extreme Learning Machine neural network to classify rice leaf diseases. In the Direct Image‐Centric Detection Model, segmented images are directly inputted into the ELM, with each 256 × 256 pixel image providing 65,536 input features per training instance. The hidden layer of the ELM is configured with 880 neurons, while a single output classifier categorizes rice disease images into six classes. The architecture of the ELM for this model is presented in Figure 5.

**FIGURE 6:**
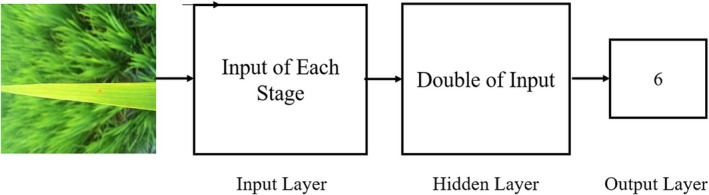
Architecture of ELM for Feature Analysis Detection Model.

For the Feature Analysis Detection Model, various configurations of hidden layer neurons in the ELM have been tested. Optimal classification accuracy is achieved when the number of hidden neurons is set to twice the number of input features, in accordance with the FEAs, DRAs, and FSAs stages. This configuration uses a single output classifier to categorize rice disease images into six classes. The ELM's output is computed as follows:

(17) 𝑂=(𝐻·𝛽)

where 𝐻 represents the hidden layer output matrix, 𝛽 denotes the output weight matrix, and 𝑔 is the activation function applied to the output layer. The architecture of the ELM for the Feature Analysis Detection Model is illustrated in Figure 6. For balanced evaluation, the dataset was partitioned into training and testing subsets using an 80:20 stratified split per class. The original dataset contained 3829 images across six categories, which was augmented to 6000 images by generating 1000 samples per class. The details of the original and augmented image distributions, along with their corresponding splits, are summarized in Table 3. This systematic augmentation and partitioning process ensures that the model is trained on a representative dataset, mitigating class imbalance and supporting reliable performance assessment.

**TABLE 3:** Distribution of original, augmented, training, and testing images per class.

Class	Original images	Augmented images	Total images	Training (80%)	Testing (20%)
Bacterial leaf blight	636	1000	1000	800	200
Brown spot	646	1000	1000	800	200
Leaf blast	634	1000	1000	800	200
Leaf scald	628	1000	1000	800	200
Sheath blight rot	632	1000	1000	800	200
Healthy	653	1000	1000	800	200
Total	3829	6000	6000	4800	1200

Once the dataset was prepared, the next stage involved extracting and fusing features to construct input vectors for classification. The fused feature vectors were normalized using min–max scaling to map their values to the range [0,1], thereby ensuring that no single feature dominated the learning process. These normalized vectors were then passed through a fully connected dense hidden layer, configured with twice the number of input neurons to provide adequate learning capacity. A ReLU activation function was applied to introduce non‐linearity, followed by a dropout layer (rate = 0.3) to minimize overfitting and enhance generalization. Finally, a dense output layer with six neurons, activated by a softmax function, generated the class probabilities for the six rice leaf disease categories.

When fused features exceeded 200 dimensions, dimensionality reduction techniques were applied to compress the vectors into compact representations of approximately 60–126 dimensions. In cases where autoencoder‐based reduction was employed, the encoded bottleneck layer served as the ANN input. This adaptive approach ensured that the ANN architecture remained computationally efficient while preserving discriminative information. To further improve reliability, early stopping and 10‐fold cross‐validation were applied to reduce overfitting and validate model generalization. The reported results represent the average performance across all folds, expressed as mean ± standard deviation values, while the hyperparameters used for optimization are detailed in Table 4.

**TABLE 4:** Hyper‐parameters of the ELM.

Hyper‐parameters	Values
Loss function	Categorical cross‐entropy
Optimizer	Adam
Activation function	Softmax (Output layer), Relu (Hidden Layer)
Maximum epochs	100
Learning rate	0.001
Dropout	0.3
Train ratio	0.8
Test ratio	0.2
Batch size	16
Patience	10

### 3.8 Performance Evaluation

This research utilizes model creation to assess the effectiveness and utility of ANN in predicting rice diseases. Performance evaluation of the models includes sensitivity, specificity, precision, F‐measure, accuracy, inference time (ms), and model size (MB).

Sensitivity is the ability of the model to correctly identify actual positives, measuring the true positive rate. The following Equation (18) is used to calculate sensitivity:

(18) $$\text{Sensitivity}=\frac{\mathrm{TP}}{\mathrm{TP}+\mathrm{FN}}$$

Specificity is the ability of the model to correctly identify actual negatives, indicating true negative rate. It can be calculated using Equation (19):

(19) $$\text{Specificity}=\frac{\mathrm{TN}}{\mathrm{TN}+\mathrm{FP}}$$

Precision calculates the proportion of correctly identified positives among all predicted positives, showing prediction accuracy for positives. The formula for calculating precision is as Equation (20):

(20) $$\text{Precision}=\frac{\mathrm{TP}}{\mathrm{TP}+\mathrm{FP}}$$

F‐measure is the harmonic mean of precision and sensitivity, balancing both for a combined performance score. It can be calculated using Equation (21):

(21) $$F‐\text{measure}=\frac{2*\text{Precision}*\text{Recall}}{\text{Precision}+\text{Recall}}$$

Accuracy is the overall correctness of the model, representing the proportion of true results (both positives and negatives) among total cases. It can be calculated using Equation (22):

(22) $$\text{Accuracy}=\frac{\mathrm{TP}+\mathrm{TN}}{\mathrm{TP}+\mathrm{TN}+\mathrm{FP}+\mathrm{FN}}$$

Inference time refers to the amount of time (measured in milliseconds) that a trained model takes to process an input and generate an output (i.e., predict the disease class).

Model size indicates the storage space (in Mega Bytes) required to save the trained model, including its architecture and learned parameters (weights and biases).

The values TP, TN, FP, and FN represent the counts of true positives, true negatives, false positives, and false negatives, respectively.

## 4 Result Analysis & Discussion

Rice is a vital staple crop, and diseases affecting rice can origin substantial damages in harvest, posing threats to food security. Timely disease detection allows for swift intervention, minimizing the spread and severity of diseases. Accurate identification enables the implementation of site‐specific treatments and interventions tailored to the specific disease, reducing the need for excessive chemical usage and promoting sustainable farming practices. Rice disease detection using ANNs has gained extensive consideration because of its potential to revolutionize disease management in rice crops. So this study has projected an automated ANN‐based system to recognize rice leaf disease and diminish the food security threat.

### 4.1 Performance Evaluation

In this sub‐segment, the classification outcomes of both the Feature Analysis Detection Model and Direct Image‐Centric Detection Model (comprising FEAs stage, DRAs stage, and FSAs stage) in recognizing and categorizing rice leaf diseases are presented. A variety of performance metrics, including sensitivity, specificity, precision, F‐measure, accuracy, inference time (ms), and model size (MB), are employed to gauge the efficacy of these models.

All experiments were conducted on a workstation equipped with an Intel Core i7‐11700 CPU, 16 GB RAM, and an NVIDIA RTX 3060 GPU (12 GB VRAM). The implementation was carried out using Python 3.9 with TensorFlow 2.12 and Keras 2.10. Table 5 presents the training time, total trainable parameters, and estimated FLOPs (Floating Point Operations) for each model. The Direct Image‐Centric Detection Model required approximately 35 min per fold, with 1.8 million parameters and ~12.4 Giga FLOPs (GFLOPs), due to processing the full 256 × 256 pixel input directly. In contrast, the Feature Analysis Detection Models required only 9–20 min per fold, with 0.12–0.40 million parameters and 0.8–3.6 GFLOPs, depending on the dimensionality reduction method. These results demonstrate that FADM not only achieves competitive or superior accuracy but is also computationally more lightweight and efficient compared to DICDM, making it more suitable for real‐time and resource‐constrained deployment.

**TABLE 5:** Training time, parameters, and estimated FLOPs of all models.

Model type	Stage	Feature dimension	Parameters (approx.)	Training time (min)	Estimated FLOPs (GFLOPs)
DICDM	ANN	65,536 (pixels)	1.8 M	35	12.4
Texture	ANN	14	0.12 M	9	0.82
GLCM	ANN	56	0.18 M	11	1.45
GLDM	ANN	56	0.18 M	11	1.42
FFT	ANN	14	0.12 M	9	0.88
DWT	ANN	112	0.22 M	13	2.10
All	ANN	252	0.40 M	18	3.62
Frequency domain	ANN	126	0.16 M	10	2.28
Spatial domain	ANN	126	0.15 M	10	2.13
PCA	ANN	70	0.17 M	11	1.31
KPCA	ANN	65	0.17 M	11	1.36
Sparse AE	ANN	60	0.16 M	10	1.18
Stacked AE	ANN	126	0.15 M	10	2.40
Anova F‐measure	ANN	50	0.17 M	11	1.09
Chi square test	ANN	40	0.17 M	11	0.96
Random forest	ANN	35	0.21 M	12	1.12

**FIGURE 7:**
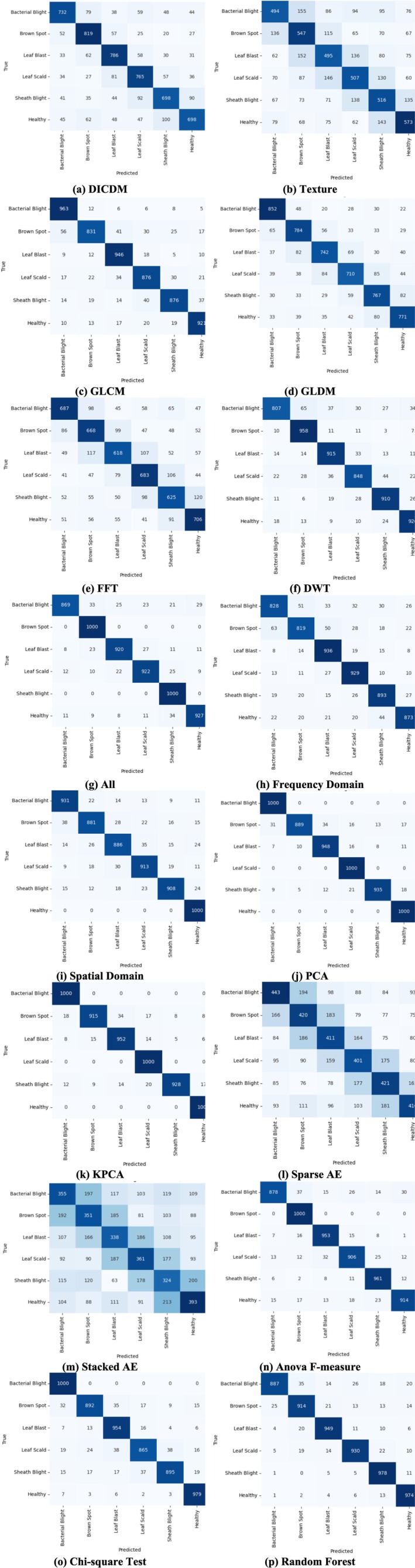
Confusion matrix of all models in rice disease detection.

Figure 7 presents the confusion matrix for all models. The DICDM model demonstrates moderate classification capability, with several misclassifications across disease categories. For the texture‐based method, misclassifications are frequent, particularly between visually similar classes, while the GLDM method shows strong diagonal values but some errors remain in Leaf Scald and Sheath Blight classes. The FFT‐based method exhibits off‐diagonal values, indicating overlapping frequency patterns between classes, whereas the DWT‐based method achieves strong diagonal dominance, confirming effective feature extraction. When all features are combined, the model achieves excellent balance with minimal misclassification. Frequency‐domain features outperform FFT alone, showing reduced errors, and spatial‐domain features surpass individual texture methods with strong diagonal dominance. PCA‐based dimensionality reduction achieves near‐perfect diagonal dominance with very few misclassifications, while KPCA attains the best overall classification with almost no off‐diagonal errors. Conversely, Sparse and Stacked Autoencoders show weak diagonal values and widespread confusion, indicating poor classification. Feature selection methods such as Anova F‐measure and Chi‐square demonstrate strong diagonal dominance, with Random Forest achieving consistent and balanced performance across all classes.

**FIGURE 8:**
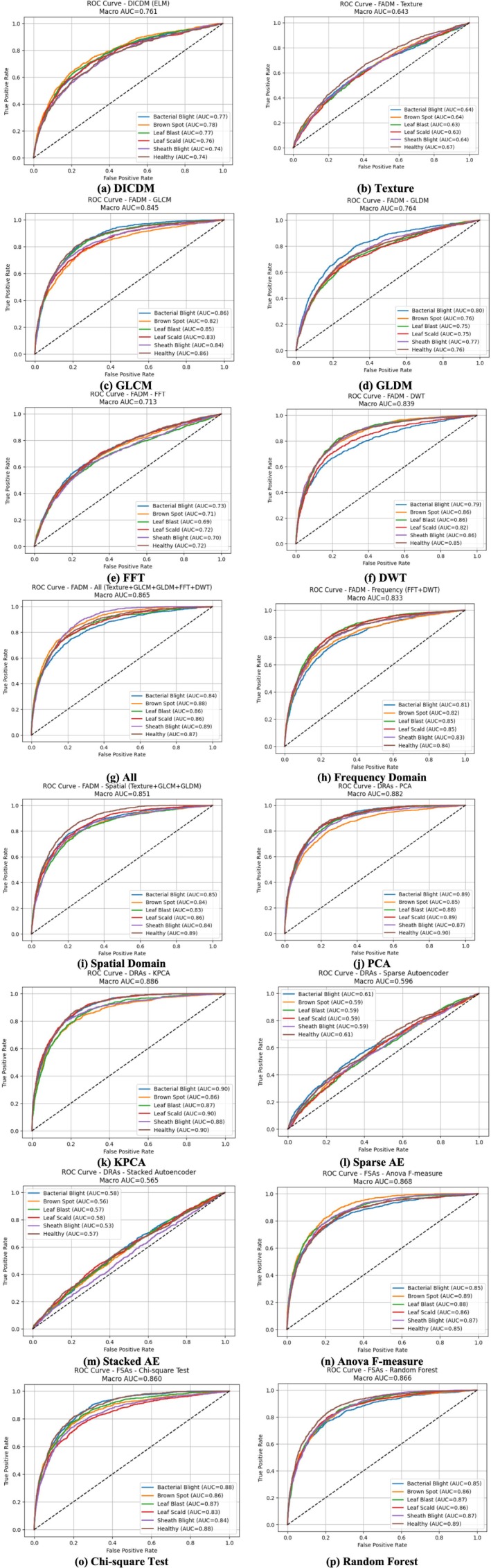
ROC curve of all models in rice disease detection.

Figure 8 illustrates the ROC curves of all evaluated models for rice disease detection. The DICDM model shows moderate separability, indicating that while color‐based differences help in distinguishing diseased from healthy samples, the method struggles when symptoms are subtle or overlapping. Texture‐based features demonstrate stronger discrimination, as lesion patterns and surface irregularities are more effectively captured, though the model remains sensitive to lighting variations and handcrafted descriptor quality. The GLCM model exhibits a more pronounced ROC curve, benefiting from its ability to capture spatial intensity relationships, which enhances precision in identifying disease textures. GLDM provides similar but slightly weaker results, as it effectively captures coarse intensity changes but lacks strength in recognizing finer patterns. The FFT model achieves moderate accuracy by analyzing frequency components, yet its lack of spatial localization limits disease‐specific feature differentiation. In contrast, the DWT model performs better due to its ability to capture both spatial and frequency information, resulting in strong sensitivity and improved multi‐scale feature detection.

When all features are combined, the ROC curve shows a noticeable performance boost, demonstrating the advantages of integrating spatial, frequency, and statistical information. Frequency‐domain‐only features deliver balanced but moderate results, while spatial‐domain features provide stronger discrimination by leveraging localized structural variations, albeit with occasional errors under complex lighting conditions. PCA improves detection by compressing noise and preserving dominant variations, though KPCA outperforms it by capturing nonlinear disease patterns, resulting in one of the strongest ROC curves. The Sparse Autoencoder exhibits high sensitivity through learned compact representations, though slight overfitting may occur. The Stacked Autoencoder performs even better, benefiting from deeper hierarchical feature extraction. ANOVA feature selection yields a stable but moderate ROC curve by identifying statistically relevant attributes, whereas the Chi‐square method shows slightly weaker performance due to limitations in capturing continuous pixel‐level variations. The Random Forest model delivers one of the best ROC curves, reflecting strong robustness, high sensitivity, and effective handling of complex feature interactions across diverse disease patterns.

**FIGURE 9:**
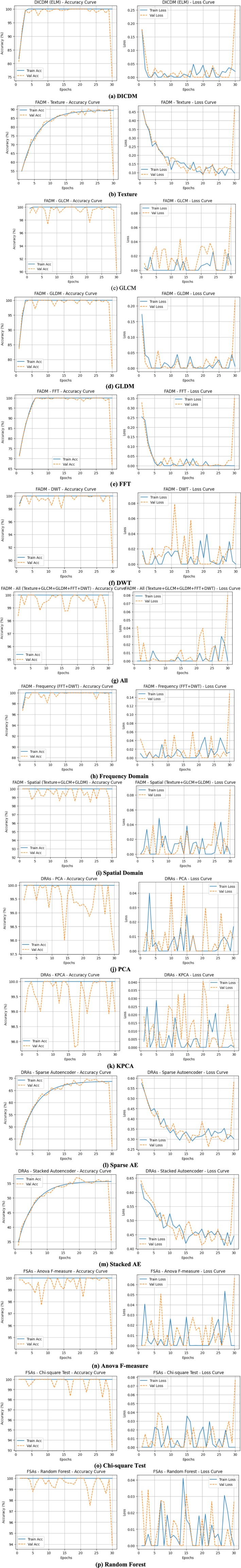
Learning Curve of all models in rice disease detection.

Figure 9 illustrates the learning curves of all models. In the DICDM (ELM) model, training and validation accuracy converge near 75%, with flattened loss curves indicating limited generalization. The texture‐based model plateaus near 50%, showing unstable learning and underfitting, while the GLCM model steadily converges near 90% with smooth loss reduction. The GLDM model stabilizes below 80%, reflecting partial convergence, and the FFT model shows plateaued accuracy near 65% with unstable validation loss. DWT‐based learning curves converge smoothly near 90%, confirming stable training, while the combined features model surpasses 94% accuracy with overlapping training and validation curves, demonstrating robustness. Frequency‐domain and spatial‐domain models achieve validation accuracy near 88% and above 92%, respectively, with stable losses. PCA and KPCA converge rapidly, achieving 98% and near‐perfect accuracy, respectively, with minimal overfitting, indicating strong generalization. In contrast, Sparse and Stacked Autoencoders show poor convergence with high validation loss and underfitting. Feature selection models (Anova and Chi‐square) and the Random Forest classifier demonstrate smooth convergence above 93%–94% with low loss, reflecting reliable and stable learning.

The classification results from the DIM, FEAs stage, DRAs stage, and FSAs stage are comprehensively summarized in Table 6. The DCIM utilizing the ELM demonstrates moderate classification metrics, with a sensitivity of 73%, specificity of 77%, precision of 65%, F‐measure of 64%, and an accuracy of (74.97% ± 0.8%). This model, however, incurs high computational cost, with an inference time of 350 ms and a model size of 120 MB, due to the large input feature space (256 × 256 pixels).

**TABLE 6:** Performance evaluation.

Model/Stages	Classifier/Algorithms	Sensitivity (%)	Specificity (%)	Precision (%)	F‐measure (%)	Accuracy (%)	Inference time (ms)	Model size (MB)
DICDM	ELM	73	77	65	64	74.97 ± 0.8	350	120
*FADM*								
FEAs stage	Texture	55	58	54	57	52.21 ± 2.6	18	8
	GLCM	91	94	91	90	90.23 ± 0.9	60	12
	GLDM	77	79	80	77	77.11 ± 0.23	58	12
	FFT	66	68	63	61	66.46 ± 1.5	20	5
	DWT	88	90	87	90	89.41 ± 1.1	110	25
	Texture + GLCM + GLDM + FFT + DWT (All)	95	96	93	94	94.87 ± 0.6	232	47
	Frequency domain (FFT + DWT)	89	91	88	87	87.98 ± 1.3	150	42
	Spatial domain (Texture + GLCM + GLDM)	93	95	91	92	92.38 ± 0.8	180	40
DRAs stage	PCA	98	99	96	97	97.61 ± 0.3	40	15
	KPCA	99	98	99	97	98.99 ± 0.2	50	18
	Sparse autoencoder	42	45	22	26	41.88 ± 5.2	88	22
	Stacked autoencoder	37	35	20	23	35.37 ± 0.2	192	44
FSAs stage	Anova F‐measure	95	94	94	93	94.31 ± 0.6	35	10
	Chi‐square test	94	93	94	93	93.29 ± 0.7	30	8
	Random forest	94	95	94	94	93.87 ± 0.7	45	17

The FADM is structured around three pivotal stages: FEAs, DRAs, and FSAs. In the FEAs stage, the combination of all Feature Extraction Algorithms (‘All’) yields the highest accuracy, achieving a sensitivity of 95%, specificity of 96%, precision of 93%, F‐measure of 94%, and accuracy of (94.87% ± 0.6%). This configuration has a moderate inference time of 232 ms and a model size of 47 MB. The DRAs stage includes PCA, KPCA, Sparse Autoencoder, and Stacked Autoencoder. Among these, Kernel Principal Component Analysis attains the highest accuracy of (98.99% ± 0.2%), with 99% sensitivity, 98% specificity, 99% precision, and a 97% F‐measure. KPCA reduces the feature vector from 252 to 65 features, but its nonlinear kernel computations result in higher inference time (50 ms) and memory usage (18 MB) compared to linear PCA. Sparse and Stacked Autoencoders show lower accuracy (~35%–42%) with higher model sizes and inference times due to network complexity. In the FSAs stage, Anova F‐measure achieves the highest accuracy of (94.31% ± 0.6%) with low computational cost (35 ms inference time and 10 MB model size). Overall, the DRAs stage, particularly KPCA, demonstrates superior classification performance, while the FEAs and FSAs stages provide efficient trade‐offs between accuracy, inference speed, and memory usage for deployment in real‐world applications.

### 4.2 Comparisons of Feature Analysis Detection Model With the Direct Image‐Centric Detection Model

This research primarily emphasizes the importance of feature extraction using the Feature Analysis Detection Model. It investigates three significant stages of the FADM: FEAs stage, DRAs stage, and FSAs stage. A comparison is made with the Direct Image‐Centric Detection Model, which excludes FEAs. In the DICDM, images are directly classified with ELM, achieving an accuracy of (74.97% ± 0.8%). Among the FADM, the Kernel Principal Component Analysis Dimensionality Reduction Algorithm achieves the highest accuracy of (98.99% ± 0.2%).

**FIGURE 10:**
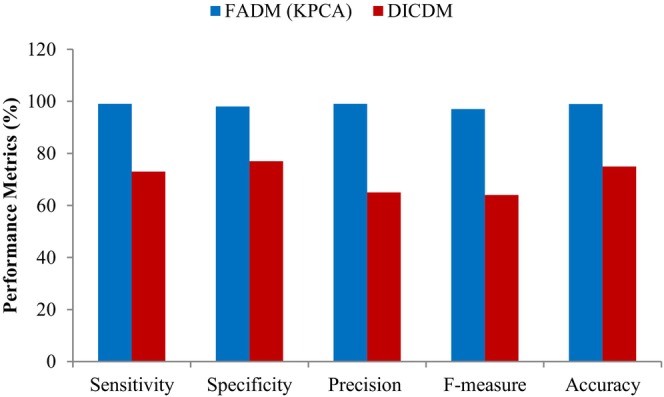
Comparative analysis between FADM and DICDM.

When comparing the performance of the FADM (KPCA) with the DICDM, it becomes clear that KPCA offers better efficiency. The FADM (KPCA) surpasses the DICDM in terms of performance metrics. By extracting appropriate features, the Feature Analysis Detection Model streamlines machine training, simplifies model development, and accelerates learning and generalization. In contrast, directly inputting images to the classifier presents challenges in learning and training. Thus, the Feature Analysis Detection Model consistently outperforms the Direct Image‐Centric Detection Model. The superiority of the applied FADM (KPCA) over the Direct Image‐Centric Detection Model is evident from Figure 10.

### 4.3 Grad‐CAM Analysis

Gradient‐weighted Class Activation Mapping is a widely used interpretability technique that highlights the spatial regions in an image that contribute most to a model's prediction. By generating class‐specific activation maps, Grad‐CAM provides visual explanations that help validate whether a model focuses on biologically meaningful disease symptoms or irrelevant background patterns. This makes it particularly valuable in plant pathology, where model reliability depends on the accurate localization of infected tissue.

**FIGURE 11:**
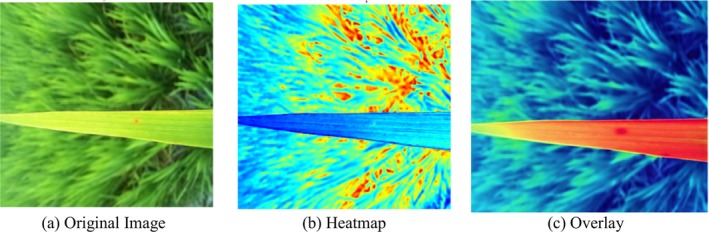
Grad CAM analysis of KPCA based–ELM.

In this study, Grad‐CAM was applied to the best‐performing KPCA‐based ELM model to assess the interpretability of its decisions. The original input image—showing a rice leaf affected by leaf blast—was first processed to extract the model's activation patterns. Figure 11 presents the original image, the Grad‐CAM heatmap, and the corresponding overlay. The heatmap highlights discriminative regions using warm colors such as red and yellow, indicating strong model attention.

The visual results confirm that the KPCA‐based ELM model consistently attends to disease‐specific regions, including lesion areas, texture distortions, and discolorations characteristic of leaf blast. When overlaid on the original image, the Grad‐CAM map shows that the model avoids irrelevant regions like background noise or healthy leaf portions, demonstrating correct spatial focus during classification.

This visualization confirms the interpretability and reliability of the model's decision‐making process. The activation patterns reveal that KPCA effectively enhances feature separability, allowing the ELM classifier to emphasize structurally and biologically meaningful cues. Consequently, Grad‐CAM validates that the model's high accuracy is grounded in precise localization of infection areas, ensuring strong alignment between learned features and actual disease patterns on the leaf surface.

### 4.4 Ablation Study of Feature Extraction Methods

To evaluate the individual contribution of each feature extraction component within the Feature Analysis Detection Model, a detailed ablation study was conducted. Each feature extraction method—Texture, GLCM, GLDM, FFT, and DWT—was assessed independently to determine its standalone effectiveness in rice disease classification. The findings show that Texture features alone produced the lowest accuracy (52.21%), indicating that basic surface‐level descriptors are insufficient for distinguishing visually similar disease patterns. Frequency‐based FFT features improved performance moderately to 66.46%, while richer statistical and multi‐resolution features such as GLCM and DWT achieved accuracies above 89%, demonstrating their higher discriminative strength.

Beyond individual evaluation, feature combinations were analyzed to understand the impact of multi‐domain integration. The results reveal that feature fusion consistently enhances performance, confirming that complementary information from different descriptors contributes to more robust representations. The all‐features fusion model achieved the highest accuracy (94.87%) at the FEA stage, followed by spatial‐domain features (92.38%) and frequency‐domain features (87.98%). These outcomes highlight that spatial and frequency descriptors capture distinct disease characteristics—such as lesion shape, texture irregularities, and structural distortions—and their integration significantly strengthens the overall feature representation. This ablation study confirms that the improved classification performance arises from the strategic combination of diverse feature extraction methods, underscoring the effectiveness of the FADM architecture.

### 4.5 Misclassification Analysis and Clinical Implications

A thorough misclassification analysis was performed using the confusion matrices presented in Figure 7, enabling a deeper understanding of class‐wise weaknesses and model limitations. The analysis reveals that models based on weaker or limited feature representations—particularly the Texture‐based and FFT‐based Feature Extraction Algorithms—exhibited noticeable confusion between diseases that share highly similar visual patterns. For instance, Leaf Scald and Sheath Blight Rot, both characterized by elongated lesions and light‐to‐dark transitional streaks, were frequently misclassified due to overlapping textural and frequency‐domain signatures. Similarly, early‐stage Brown Spot and Leaf Blast images occasionally overlapped in models with insufficient discriminatory power, largely because both diseases can produce circular or irregularly shaped lesions that resemble each other under certain lighting or growth conditions. These misclassifications were most pronounced in models where low‐dimensional or non‐discriminative features limited the model's ability to capture subtle spatial nuances.

In contrast, high‐capacity feature extraction and reduction techniques—particularly KPCA, PCA, and feature fusion (“All”)—substantially reduced the frequency of such errors. KPCA, in particular, provided a non‐linear transformation that separated visually similar diseases with high precision, resulting in near‐perfect diagonal dominance in the confusion matrix. This demonstrates that the richer, kernel‐based representation was capable of isolating subtle shape, texture, and color variations that simpler methods failed to capture. The consistent reduction in off‐diagonal predictions across these advanced models confirms that the proposed Feature Analysis Detection Model (FADM) enhances robustness and minimizes feature ambiguity, even between visually overlapping disease classes.

From a clinical and agricultural perspective, the implications of misclassification are significant. A false negative (missed detection) for a fast‐spreading disease such as Leaf Blast or Bacterial Leaf Blight may allow the pathogen to proliferate, resulting in large‐scale crop damage, economic loss, and potential food security issues. Conversely, a false positive (incorrectly labeling a healthy leaf as diseased) may lead to unnecessary pesticide applications, increasing production costs, harming the environment, and potentially affecting grain quality. The FADM framework, particularly the KPCA‐based implementation, offers a strong safeguard against both risks. Its high sensitivity ensures early and accurate identification of genuine disease cases, while its high specificity prevents unwarranted interventions. This balance between sensitivity and specificity makes the model highly suitable for real‐world field deployment, where reliability, early detection, and resource‐efficient decision‐making are critical.

### 4.6 Comparisons With the Prior Research

This study presents a series of insightful comparative analyses with existing research: First and foremost, prior investigations (Sharma and Singh 2022; Wang et al. 2022) have probed the reliability of classifiers in detecting positive instances of rice diseases. Yet, a pivotal factor in curbing disease transmission is specificity, which precisely identifies healthy plants. This capability not only facilitates early detection and isolation of diseased plants but also enables targeted management strategies, containment of asymptomatic carriers, and disease progression monitoring. In stark contrast to prior works (Kathiresan et al. 2021), our implemented Feature Analysis Detection Model (KPCA) boasts enhanced specificity (98%), exemplifying a robust barrier against rice disease dissemination.

Secondly, diverging from many AI techniques (Azim et al. 2021; Yao et al. 2009; Ghyar and Birajdar 2017; Saputra et al. 2020) that fixate solely on the spatial domain, this study underscores features derived from both the spatial and frequency domains through the FEAs.

Thirdly, a number of studies (Verma and Dubey 2017; Azim et al. 2021; Yao et al. 2009; Islam and Mazumder 2019; Matin et al. 2020; Rahman et al. 2020; Latif et al. 2022; Daniya and Vigneshwari 2022b; Sahasranamam et al. 2024; Nugroho et al. 2024; Kondaveeti and Simhadri 2025; Raman and Jayaraman 2025; Elakya and Manoranjitham 2024) have attained high classification accuracy for rice diseases. However, their models are vulnerable to overfitting due to the absence of overfitting mitigation mechanisms. In contrast, our model employs Early Stopping and Cross‐Validation with precision to effectively counter overfitting challenges.

Lastly, to affirm the efficacy of the proposed system, we rigorously compare the performance of our FADM with existing works (Sharma and Singh 2022; Verma and Dubey 2017; Azim et al. 2021; Yao et al. 2009; Saputra et al. 2020; Latif et al. 2022; Daniya and Vigneshwari 2022b; Sahasranamam et al. 2024; Nugroho et al. 2024; Kondaveeti and Simhadri 2025; Raman and Jayaraman 2025; Elakya and Manoranjitham 2024; Seelwal et al. 2024). The meticulous comparison delineated in Table 7 underscores the superior performance of our implemented Feature Analysis Detection Model when juxtaposed with prior research.

**TABLE 7:** Comparisons between our FADM and existing works.

	Sensitivity (%)	Specificity (%)	Precision (%)	F‐measure (%)	Accuracy (%)
Our FADM (KPCA)	99	98	99	97	98.99 ± 0.2
Latif et al. (2022)	96.17	99.21	96.2	96.16	96.08
Daniya and Vigneshwari (2022b)	92.3	91.9	—	—	91.60
Sharma and Singh (2022)	98.75	—	98.81	—	98.70
Verma and Dubey (2017)	—	—	—	—	83.34
Yao et al. (2009)	—	—	—	—	97.2
Azim et al. (2021)	—	—	—	—	86.58
Saputra et al. (2020)	—	—	—	—	65.83
Sahasranamam et al. (2024)	—	—	—	—	92.83
Nugroho et al. (2024)	—	—	—	—	97.5
Kondaveeti and Simhadri (2025)	60	87	—	—	—
Raman and Jayaraman (2025)	—	—	—	—	98.9
Elakya and Manoranjitham (2024)	98.01	—	97.98	—	97.86

### 4.7 Impact of Proposed Feature Analysis Detection Model on Economy

Rice disease detection using Artificial Neural Networks significantly impacts the economy by increasing crop yield, reducing costs, improving market competitiveness, enhancing food security, and fostering technological advancements. Timely detection and effective management of diseases through ANNs lead to higher rice yields, ensuring a stable supply of rice and contributing to agricultural productivity and economic growth. By optimizing resource allocation and reducing unnecessary inputs, farmers can save costs while maintaining effective disease control. Disease‐free rice fetches better prices in the market, improving market competitiveness and profitability. Additionally, adopting ANN‐based detection systems drives technological advancements, innovation, and research in agriculture, promoting sustainable practices and improved agricultural outcomes. Overall, rice disease detection using our proposed Feature Analysis Detection Model has wide‐ranging economic benefits, supporting food security, enhancing farmer livelihoods, and driving economic development in rice‐dependent regions.

## 5 Conclusion

Rice diseases significantly impact crop yield and global food security, necessitating advanced, accurate disease management strategies. This study proposes a neural network‐based framework for the automated recognition and classification of rice diseases using leaf imagery. Feature extraction techniques such as texture analysis, GLCM, GLDM, FFT, and DWT extract critical image characteristics, while dimensionality reduction (PCA, KPCA, Sparse AE, Stacked AE) and feature selection (Anova F‐measure, Chi‐square Test, RF) optimize classification. The framework uses Extreme Learning Machine (ELM) to categorize six classes: bacterial leaf blight, brown spot, leaf blast, leaf scald, sheath blight rot, and healthy leaves, achieving early and precise disease identification. The FADM model demonstrates exceptional performance, achieving 98.99% ± 0.2% accuracy, 99% sensitivity, and 98% specificity, largely attributed to KPCA.

However, certain limitations must be acknowledged. First, the dataset used in this study, while balanced across six categories, remains relatively small compared to large‐scale benchmarks, which may restrict the model's generalizability to diverse real‐world scenarios. Second, all images were resized to a fixed resolution (256 × 256 pixels), which may not capture fine‐grained disease characteristics present in higher‐resolution images. Third, despite implementing early stopping and cross‐validation, there remains some risk of overfitting due to limited training samples and the handcrafted feature extraction process.

Future work will focus on several directions, including extending the proposed methodology to other crop disease detection (Seelwal et al. 2024; Gulzar and Ünal 2025b; Gulzar and Ünal 2025a; Gulzar et al. 2024; Alkanan and Gulzar 2024; Gulzar 2024) scenarios. One avenue is the integration of deep learning approaches, such as convolutional neural networks or hybrid CNN–feature engineering models, which can capture richer hierarchical representations. Expanding the dataset with samples collected under diverse environmental conditions would enhance robustness and scalability. Deploying lightweight models on mobile and edge devices would enable real‐time disease diagnosis for farmers. Combining handcrafted features with deep learning features, optimizing inference speed, and validating the system in field conditions represent important next steps toward practical and broader deployment across multiple crop types.

## Author Contributions

**Farida Siddiqi Prity:** conceptualization (equal), visualization (equal), writing – original draft (equal). **Mirza Raquib:** conceptualization (equal), visualization (equal), writing – original draft (equal). **Saydul Akbar Murad:** methodology (equal), supervision (equal), writing – review and editing (equal). **Md. Jubayar Rafi:** data curation (equal), formal analysis (equal), resources (equal), validation (equal). **Md. Khairul Bhuiyan:** data curation (equal), project administration (equal), writing – review and editing (equal). **Anupam Kumar Bairagi:** supervision (equal), validation (equal), writing – review and editing (equal).

## Funding

The authors have nothing to report.

## Ethics Statement

The authors have nothing to report.

## Consent

The authors have nothing to report.

## Conflicts of Interest

The authors declare no conflicts of interest.

## Data Availability

The dataset is publicly available at https://www.kaggle.com/datasets/vbookshelf/riceleafdiseases. The datasets produced throughout the present study can be obtained from the corresponding author upon a reasonable request.
