# Correction to “Neural Network‐Based Study for Rice Leaf Disease Recognition and Classification: A Comparative Analysis Between Feature‐Based Model and Direct Imaging Model”

**DOI:** 10.1002/fsn3.71440

**Published:** 2026-01-09

Prity, F. S., M. Raquib, S. A. Murad, M. J. Rafi, M. K. Bhuiyan, and A. K. Bairagi. 2025. “Neural Network‐Based Study for Rice Leaf Disease Recognition and Classification: A Comparative Analysis Between Feature‐Based Model and Direct Imaging Model.” *Food Science & Nutrition* 13, no. 12: e71350. https://doi.org/10.1002/fsn3.71350

In the originally published version, there are spelling errors in two authors’ names:

Md. Jubayar Rafi should be corrected to Md. Jubayar Alam Rafi and Md. Khairul Bhuiyan should be corrected to Md. Khairul Bashar Bhuiyan.

Affiliation 2 had the incorrect department and city. It should be corrected to: Department of Computer and Communication Engineering, International Islamic University Chittagong, Chittagong, Bangladesh.

The original article has been updated accordingly.

We apologize for this error.

